# *extra macrochaetae* , encoding *Drosophila* Id, controls apical cell shape in the hindgut epithelium

**DOI:** 10.17912/micropub.biology.000526

**Published:** 2022-03-17

**Authors:** Tomoki Ishibashi, Kenji Matsuno

**Affiliations:** 1 Department of Biological Sciences, Graduate School of Science, Osaka University, Toyonaka, Osaka 560-0043, Japan; 2 Laboratory for Physical Biology, RIKEN Center for Biosystems Dynamics Research, Kobe 650-0047, Japan

## Abstract

Inhibitor of DNA-binding (Id) transcription factor regulates the balance of cell differentiation and proliferation and is involved in organ morphogenesis in various species. Previously, we revealed that *extra macrochaetae* ( *emc* ), encoding the only Id protein in *Drosophila* , controls chirality of cell shape in hindgut epithelium. Here, to further understand functions of *emc* in cell-shape regulations, we analyzed apical cell shape in the hindgut epithelium of *emc* mutant embryos. We found that *emc* mutants showed expansion of their apical surface, but no abnormalities in cell differentiation and proliferation. Therefore, our results demonstrate that Id can control cell morphology without affecting specification and propagation of cells.

**Figure 1. The apical area of epithelial cells and the epithelial-tube diameter increased in the embryonic hindgut of an emc mutant.**
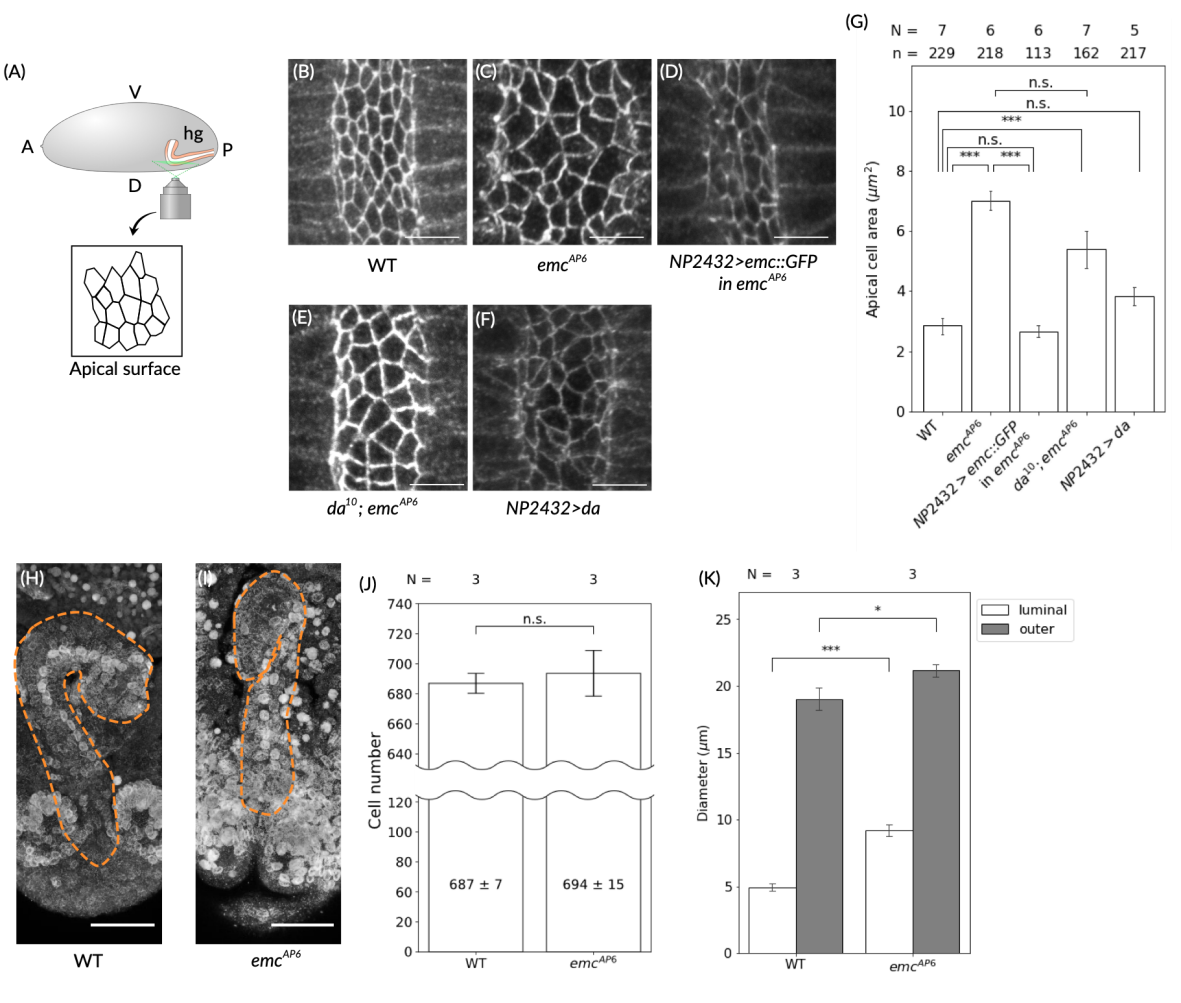
(A) A diagram showing imaging of the *Drosophila* embryonic hindgut epithelium (orange) under a confocal microscope. The embryo (shown in gray) is viewed from the right side (A: anterior; P: posterior; D: dorsal; V: ventral). The hindgut (hg) and its epithelium are shown in white and orange, respectively. We imaged the dorsal side of the hindgut luminal (apical) surface (the lateral view is indicated by a green line) and determined apical-surface cell boundaries. (B-F) Representative images showing apical-surface cell boundaries at stage 12 (before hindgut rotation) in the following genotypes: (B) wild-type (WT), (C) an *emc ^AP6^* homozygote ( *emc ^AP6^* ), (D) an *emc ^AP6^* homozygote overexpressing *UAS-emc* :: *GFP* ( *NP2432>emc* :: *GFP* in *emc ^AP6^* ), (E) a double homozygote of *da ^10^* and *emc ^AP6^* ( *da ^10^* ; *emc ^AP6^* ), and (F) wild-type overexpressing *UAS-da* ( *NP2432>da* ). Scale bars: 5 μm. (G) A bar graph showing the mean apical cell-surface area in the hindgut of stage 12 embryos with the indicated genotypes (described in B-F). The number of embryos (N) and the number of cell boundaries (n) analyzed is shown above each genotype. ***: *p* < 0.001; n.s: not significant; significance was determined by Tukey's multiple comparison test with a 5% significance level. (H) Typical images of the hindgut nulcei, stained with an anti-Lamin C antibody, in the WT embryo at stage 13 (after hindgut rotation) and (I) in the *emc ^AP6^* homozygous ( *emc ^AP6^* ) embryo at stage 13 (corresponding to the period when the WT hindgut is completing its rotation). The dotted orange lines indicate the outline of the hindgut). Scale bars: 25 μm. (J, K) Bar graphs showing (J) the number of cells (values show mean ± standard error) in the hindgut epithelium and (K) the mean luminal (white) and outer (grey) diameters of the hindgut in WT and *emc ^AP6^* embryos at stage 13. The number of embryos analyzed (N) is shown above each genotype. ***: *p* < 0.001; *: *p* < 0.05; n.s: not significant; significance was determined by likelihood-ratio test with a 5% significance level. Error bars indicate standard error.

## Description

The basic helix-loop-helix (bHLH) transcription factor is a representative class of transcriptional regulators conserved in a wide range of eukaryotes (Gyoja, 2017). The bHLH proteins form homo- or heterodimers and bind DNA, thereby controlling cell proliferation and differentiation. The first reported bHLH proteins were found to bind E-box DNA sequences and were thus named E proteins (Murre *et al* . 1989). E proteins are essential for mesodermal and neural development in various species (Wang & Baker, 2015).

Another class of HLH proteins, known as antagonists of bHLH transcription factors, are called Inhibitor of DNA-binding (Id) proteins. Id proteins form a heterodimer with bHLH proteins and thereby inhibit their ability to bind DNA (Murre *et al* . 1989). The balance of E and Id proteins is important for regulating cell proliferation and differentiation (Wang & Baker, 2015). *Drosophila* also has single orthologous genes encoding the E and Id proteins, *daughterless* ( *da* ) and *extra macrochaetae* ( *emc* ), respectively (Caudy *et al* . 1988a; Caudy *et al* . 1988b; Ellis *et al* . 1990; Garrell & Modolell, 1990). Thus, the functions of the E and Id proteins are relatively easy to study in *Drosophila* compared with those in vertebrate model organisms, which have multiple *E* and *Id* genes (Gyoja, 2017). In *Drosophila* , Da forms a homodimer or a heterodimer with bHLH proteins and upregulates the expression of downstream genes, while Emc antagonizes Da by binding it in an inactive heterodimer (Garrell & Modolell, 1990; Murre *et al* . 1989). The regulation of cell-fate specification and organ morphogenesis through the balance between Emc and Da is well studied (Celis *et al* . 1995). However, the function of Da and Emc in controlling cellular morphology is less clear. We previously revealed that the suppression of Da by Emc is required for forming cell chirality in hindgut epithelial cells, which consequently drives the left-right (LR)-asymmetric development of the hindgut (Ishibashi *et al* . 2019; Ishibashi *et al* . 2020). In this study, we further explored the roles of Da and Emc in regulating epithelial cell morphology.

Here, we analyzed the morphology of hindgut epithelial cells in *Drosophila* embryos in which *emc* and *da* were genetically modulated (Fig. 1 A). The hindgut epithelial tube is formed with monolayer epithelium whose luminal side corresponds to the apical surface of each epithelial cell. We found that the area of the apical surface was significantly larger in *emc ^AP6^* homozygotes than in the wild-type embryos at stage 12, which corresponds to the period just before hindgut rotation begins in the wild-type embryo (Fig. 1 B, C, G; *p* < 0.001), and that the lumen of the hindgut was markedly larger in *emc ^AP6^* homozygotes than in wild-type embryos in all cases examined (N=7) (Fig. 1 C). The tissue-specific overexpression of *UAS-emc::GFP* in the hindgut epithelium, driven by *NP2432* , significantly rescued the apical-area expansion in *emc ^AP6^* homozygotes ( *NP2432>emc::GFP* in *emc ^AP6^* ) at stage 12, demonstrating that the expansion of the apical area can be attributed to the absence of *emc* function (Fig. 1 D, G; *p* < 0.001), and also rescued luminal expansion in all cases examined (N=6) (Fig. 1 D). We previously revealed that the *emc* mutation did not affect the tissue-specification of the hindgut (Ishibashi *et al* . 2019). Thus, the expansion of the apical area in *emc ^AP6^* mutants may not be associated with cell-fate changes in the hindgut epithelial cells.

We previously showed that a proper Da–Emc balance is essential for the LR-asymmetric development of the embryonic hindgut (Ishibashi *et al* . 2019). The embryonic hindgut forms first as a bilaterally symmetric structure at early stage 12, and its anterior part curves toward the ventral side of the embryo (Hayashi & Murakami, 2001). At this stage, the hindgut epithelial cells are intrinsically chiral. The subsequent dissolution of cell chirality induces the 90° counterclockwise rotation of the hindgut, causing the anterior part of the hindgut to curve to the right at stage 13 in wild-type embryos (Fig. 1 H) (Taniguchi *et al* . 2011). Our previous study revealed that in *emc ^AP6^* homozygotes, hindgut LR symmetry becomes randomized when cell chirality disappears at stage 13 (Fig. 1 I) (Ishibashi *et al* . 2019; Ishibashi *et al* . 2020). This LR randomization and loss of cell chirality associated with homozygous *emc ^AP6^* was completely suppressed in combination with homozygous *da ^10^* , because *da* hyperactivation causes LR randomization in *emc* mutants (Ishibashi *et al* . 2019; Ishibashi *et al* . 2020). In contrast, we here found that the expansion of the apical area associated with homozygous *emc ^AP6^* was not significantly suppressed in combination with homozygous *da ^10^* at stage 12 ( *da ^10^ ; emc ^AP6^* in Fig. 1 E, G; *p* > 0.05). Thus, the expansion of the apical area in the *emc ^AP6^* homozygote is independent of *da* . This is further supported by our finding that although tissue-specific *da* overexpression in the hindgut epithelium of otherwise wild-type embryos rescued the condition of the *emc* mutant hindgut, it did not increase the apical surface area of the hindgut epithelium, whereas our previous study revealed that such *da* overexpression induces random LR asymmetry of the hindgut and the loss of cell chirality ( *NP2432>da* in Fig. 1 F, G) (Ishibashi *et al* . 2019; Ishibashi *et al* . 2020). These data indicate that *emc* is required for regulating apical area independently of *da* .

Emc/Id proteins control cell proliferation in various species (Wang & Baker, 2015). Therefore, the expansion of the apical area in the *emc* homozygote may be indirectly caused by changes in the number of hindgut epithelial cells. To test this possibility, we visualized the nuclei in the hindgut epithelium by immunostaining with an anti-Lamin C antibody, since *Lamin C* is expressed specifically in the epithelium of the embryonic hindgut at stage 13 (Fig. 1 H, I) (Riemer *et al* . 1995), and manually counted the nuclei in the hindgut epithelium. The number of cells in the hindgut epithelium stays the same during hindgut rotation (stage 12-13). We found that the number of nuclei in the hindgut epithelium was almost the same in wild-type and *emc ^AP6^* homozygote embryos (Fig. 1 J), suggesting that the expansion of the apical area in the *emc ^AP6^* homozygote is caused by morphological changes in the hindgut epithelial cells, not by a change in the number of cells. We then speculated that such structural abnormalities in individual cells might be coupled with alterations in the overall structure of the hindgut. To examine this possibility, we measured the luminal and outer diameters of the hindgut tube in wild-type embryos and *emc* homozygotes. We found that the mean luminal and outer diameters of the hindgut were significantly larger in the *emc ^AP6^* mutants (Fig. 1 K), demonstrating that the hindgut tube became thicker in the *emc ^AP6^* mutant compared with the wild-type embryo.

Taken together, we concluded that *emc* regulates the apical area of hindgut epithelial cells independently of *da* . Our results also show that *emc* controls the shape of hindgut epithelial cells, but not their proliferation, to produce proper hindgut architecture (such as luminal and outer diameters). However, the mechanisms by which *emc* affects the shape of hindgut epithelial cells and the global architecture of the hindgut remains unknown.

## Methods

Fly stocks: we used *Canton-S* as a wild-type line. We used *emc ^AP6^* as an amorphic allele of *emc* (Bloomington #36544) and *da ^10^* as an amorphic allele of *da* (Bloomington #5531) (Ellis, 1994; Wülbeck *et al* . 1994). We used the following UAS lines: *UAS-emc::GFP* (Popova *et al* . 2011) and *UAS-da* (Bloomington #51669) (Giebel *et al* . 1997). As a hindgut epithelium-specific *gal4* driver, we used *NP2432* (Kyoto DGRC #104201) (Hayashi *et al* . 2002). Mutations on the second chromosome were balanced with *CyO, P{en1}wg ^en11^* . Mutations on the third chromosome were balanced with TM3, P{ftz-lacZ.ry ^+^ }TM3, Sb ^1^ ry ^*^ , or *TM6B, P{iab-2(1.7)lacZ}6B, Tb ^1^* . All genetic crosses were carried out at 25 °C on a standard *Drosophila* culture medium.

*Drosophila* embryos were collected and dechorionated in 3% hypochlorous acid solution. The dechorionated embryos were fixed with 6% paraformaldehyde in PBS (130 mM NaCl, 7 mM Na _2_ HPO _4_ , and 3 mM NaH _2_ PO _4_ ) for 30 minutes and the vitelline membrane was removed by washing in 100% methanol. The embryos were fixed in 100% methanol at −20 °C.

The fixed embryos were treated with 2% Block Ace (Yukijirushi) in PBT (130 mM NaCl, 7 mM Na _2_ HPO _4_ , 3 mM NaH _2_ PO _4_ , and 0.1 % (w/v) Triton-X-100). The embryos were then washed with PBT and incubated in the appropriate primary antibody solutions in Can Get Signal Solution B (TOYOBO) in 2 mL tubes (Eppendorf) at room temperature for 3 hr or at 4 °C overnight. The embryos were then washed with PBT and incubated in the appropriate secondary antibody solutions at room temperature for 3 hr or at 4 °C overnight. After washing, the embryos were dehydrated with ethanol and mounted with clearing reagent (methyl salicylate). The following primary antibodies were used at the dilutions indicated: mouse anti-phosphotyrosine antibody (PY20) (1:1,000, BD Biosciences), chicken anti-β-galactosidase antibody (1:500, Abcam), and mouse anti-Lamin C antibody (1:1000, DSHB). We used the following secondary antibodies at the indicated dilutions: anti-chicken IgY-Alexa 488 (1:1,000, Jackson ImmunoResearch) and anti-mouse IgG-Cy3 (1:1000, Jackson ImmunoResearch). We processed images of the immunostained hindgut using Adobe Photoshop CS3 (Adobe Systems) or Adobe Illustrator CS3 (Adobe Systems).

To analyze apical cell size, we obtained images of the apical cell boundaries at stage 12. We manually defined the cell boundaries and measured the apical cell area with ImageJ Fiji (Schindelin *et al* . 2012).

To count nuclei in the hindgut, we stained stage 13 embryos with anti-Lamin C antibody. Images of the whole hindgut were captured with an LSM880 confocal microscope (Carl Zeiss) in wild-type and *emc ^AP6^* homozygous embryos. We manually selected the centroids of Lamin C-positive nuclei in three-dimensional space using ImageJ Fiji (Schindelin *et al* . 2012) and calculated the number of nuclear centroids.

To measure the luminal and outer perimeters of the hindgut, we visualized hindgut cell boundaries by immunostaining with anti-PY20 antibody. Images of the whole hindgut were captured with an LSM880 confocal microscope (Carl Zeiss) in WT and *emc ^AP6^* homozygous embryos at stage 13. We examined serial optical sections located halfway between the anterior and posterior ends of the hindgut, and selected a representative image showing the maximal diameter of the middle portion along the gut tube. We then measured the luminal and outer perimeters manually.
